# Three tRNA nuclear exporters in *S. cerevisiae*: parallel pathways, preferences, and precision

**DOI:** 10.1093/nar/gkac754

**Published:** 2022-09-13

**Authors:** Kunal Chatterjee, William A Marshall, Anita K Hopper

**Affiliations:** Department of Molecular Genetics, Ohio State University, Columbus, OH 43235, USA; Center for RNA Biology, Ohio State University, Columbus, OH 43235, USA; Department of Molecular Genetics, Ohio State University, Columbus, OH 43235, USA; Department of Molecular Genetics, Ohio State University, Columbus, OH 43235, USA; Center for RNA Biology, Ohio State University, Columbus, OH 43235, USA

## Abstract

tRNAs that are transcribed in the nucleus are exported to the cytoplasm to perform their iterative essential function in translation. However, the complex set of tRNA post-transcriptional processing and subcellular trafficking steps are not completely understood. In particular, proteins involved in tRNA nuclear export remain unknown since the canonical tRNA nuclear exportin, Los1/Exportin-t, is unessential in all tested organisms. We previously reported that budding yeast Mex67-Mtr2, a mRNA nuclear exporter, co-functions with Los1 in tRNA nuclear export. Here we employed *in vivo* co-purification of tRNAs with endogenously expressed nuclear exporters to document that Crm1 also is a *bona fide* tRNA nuclear exporter. We document that Los1, Mex67-Mtr2 and Crm1 possess individual tRNA preferences for forming nuclear export complexes with members of the 10 families of intron-containing pre-tRNAs. Remarkably, Mex67-Mtr2, but not Los1 or Crm1, is error-prone, delivering tRNAs to the cytoplasm prior to 5′ leader removal. tRNA retrograde nuclear import functions to monitor the aberrant leader-containing spliced tRNAs, returning them to the nucleus where they are degraded by 3′ to 5′ exonucleases. Overall, our work identifies a new tRNA nuclear exporter, uncovers exporter preferences for specific tRNA families, and documents contribution of tRNA nuclear import to tRNA quality control.

## INTRODUCTION

tRNAs perform the essential function of delivering amino acids, specified by mRNA codons, to ribosomes during protein synthesis. In addition to this canonical role in translation, tRNAs have several other ancillary functions (1). Therefore, defects in tRNA biosynthesis cause numerous diseases such as cancer, and metabolic and neuromuscular disorders (2).

tRNA biogenesis involves a multitude of processing and modification steps that occur at different cellular locations and involve multiple subcellular trafficking steps. Some of the proteins that participate in these steps remain unknown. Nascent tRNAs contain 5′ leader and 3′ trailer sequences that are removed prior to export of tRNAs from the nucleus to the cytoplasm (1,3,4). After removal of the 5′ leader and the 3′ trailer, the CCA sequence is then added to the 3′ end (5). Further, ∼12% of the tRNA nucleotides are post-transcriptionally modified by enzymes that reside in either the nucleus or the cytoplasm (1). In archaea and eukaryotes, a subset of the tRNA genes contain introns that are removed by tRNA splicing endonucleases. In the yeast *Saccharomyces cerevisiae*, 10 of the 42 tRNA gene families contain introns. In budding yeast, fission yeast, trypanosomes, and plants, intron removal occurs in the cytoplasm after tRNA nuclear export, but in vertebrates tRNA splicing occurs in the nucleus (6–13).

tRNAs move bidirectionally between the nucleus and the cytoplasm (14–19). The first nuclear export step is termed primary tRNA nuclear export. Cytoplasmic tRNAs return to the nucleus by the process termed tRNA retrograde nuclear import and, under appropriate environmental conditions, imported tRNAs return, once again, to the cytoplasm in the tRNA nuclear re-export step. In yeast, primary tRNA nuclear export can be distinguished from tRNA re-export for tRNAs that are encoded by intron-containing genes because tRNAs undergoing primary export possess introns whereas tRNAs undergoing re-export do not since the introns are spliced prior to retrograde nuclear import.

Los1 (Exportin-t in vertebrates, Xpo-t in fission yeast, and PAUSED in plants) which functions in both the tRNA primary and re-export steps, was the first, and for a long time, the only known tRNA nuclear exporter (20–26). However, there is considerable evidence that there are additional tRNA nuclear exporters in most eukaryotes. First, in all tested organisms, including haploid human cancer cells, *LOS1* and its homologs are unessential (21,27–31). Second, *Drosophila melanogaster* lacks a Los1 homologue (32). Further, the *Trypanosome brucei* Los1 orthologue, TbXpo-t, does not appear to function in tRNA nuclear export of tRNA^Tyr^, the only intron-containing tRNA, and it is also unessential (33). Finally, there is a pool of spliced tRNAs that contain 5′ leaders in budding yeast and vertebrate cells (34,35). This pool of tRNAs in yeast results from precocious tRNA nuclear export prior to end maturation, followed by splicing in the cytoplasm. However, biochemical and structural studies have documented that Xpo-t/Exportin-t only interacts with tRNAs possessing mature 5′ and 3′ termini (6,36–39). Thus, nuclear export of pre-tRNAs with 5′ leaders requires Los1/Exportin-t-independent nuclear exporter(s).

Los1-independent tRNA nuclear exporters have since been discovered. The Exportin-5 family (Msn5 in yeast, Exportin-5 in vertebrates, and HASTY in plants) which exports noncoding RNAs, such as pre-microRNAs to the cytoplasm, also participates in tRNA nuclear export (38,40–44). However, budding yeast Msn5 preferentially binds spliced and aminoacylated tRNAs and therefore it functions in the tRNA re-export step, rather than the primary tRNA nuclear export (38,44).

Employing the yeast gene deletion collection and two temperature sensitive mutant libraries, we screened for strains that accumulated unspliced tRNAs as a proxy for defects in the primary tRNA nuclear export step (45). We discovered that the Mex67-Mtr2 heterodimeric (NXF1-NXT1 in metazoans) mRNA nuclear exporter co-functions with Los1 in both the primary and re-export steps (46). Surprisingly, a mere five-fold overexpression of Mex67–Mtr2 substitutes for Los1 in *los1Δ* cells by suppressing the tRNA nuclear export defect for all 10 intron-containing tRNA families. Moreover, employing an *in vivo* chemical cross-linking and co-immunoprecipitation assay, we demonstrated that over-expressed Protein A-tagged Mex67 binds both unspliced and spliced tRNAs, thus documenting its function as a tRNA nuclear exporter (46).

In the same genome-wide screen, Crm1 (Xpo-1 in *Schizosaccharomyces pombe* and humans) was also implicated in tRNA nuclear export (45). Crm1 functions in nuclear export of proteins containing a leucine rich motif, some of which function in nuclear export of rRNAs, snRNAs, mRNA and microRNAs (47,48). At the nonpermissive growth temperature, *crm1-1* mutant cells accumulate end-processed, intron-containing tRNAs in the nucleus—a hallmark of defective tRNA nuclear export (45). Moreover, *los1Δ* *crm1-1* double mutant cells exhibit synthetic growth defects, indicating that Crm1 has overlapping functions with Los1 (45). Finally, a recent study demonstrated that nuclear re-export of spliced tRNA^Phe^_GAA_ is dependent upon Mex67 and Crm1, rather than the canonical Los1 or Msn5 nuclear exporters (49). However, it remained unknown whether Crm1 functions in tRNA nuclear export by forming nuclear export complexes with tRNAs, or, instead, if it functions indirectly in tRNA nuclear export.

In this study, we verified Crm1 to be a *bona fide* tRNA nuclear exporter by documenting that endogenously expressed Crm1 forms *in vivo* nuclear export complexes with intron-containing tRNAs. We conclude that there are at least three tRNA nuclear exporters in yeast that function in primary tRNA export: Los1, Mex67-Mtr2 and Crm1. We further document that these nuclear exporters possess different tRNA family preferences. Finally, we show that whereas Los1 and Crm1 have high fidelity in exporting tRNAs with mature 5′ termini, Mex67 is able to bind pre-tRNAs with 5′ unprocessed leaders and thereby Mex67 can export tRNAs to the cytoplasm prior to end-processing. After intron removal in the cytoplasm, the spliced 5′ leader-containing ‘aberrant’ tRNAs can return to the nucleus via retrograde import where 3′ to 5′ nucleases function in their destruction.

## MATERIALS AND METHODS

### *In vivo* co-immunoprecipitation assay

The protocol was similar to that previously described (46), with some modifications (detailed in Supplementary materials).

### Western analyses

The antibodies and the concentrations used in western analyses were as follows: anti-GFP (Mouse; Abcam) was used at 1:1000 dilution, anti-Ran (gift from Dr P. Belhumeur, Univ. Montreal, Canada) at 1:10 000 dilution. Secondary antibodies used were goat anti-mouse IgG antibodies (IRDye^®^ 680 CW, LI-COR) at 1:1000 dilution and goat anti-rabbit IgG antibodies (IRDye^®^ 800 CW, LI-COR) at 1:10 000 dilution. The IR signals were visualized by LI-COR ODYSSEY platform machine and images were obtained using the LI-COR Image Studio™ software.

### RT-PCR

The protocol followed to assess tRNA species co-immunoprecipitating with GFP-tagged nuclear exporters was similar to that described in (38,46) with some modifications. Details are provided in the Supplementary materials.

### RT-qPCR

DNase treatment and first strand cDNA synthesis were the same as described for RT-PCR. RT-qPCR reactions were carried out using iTaq Universal SYBR Green super mix (Biorad) according to manufacturer's protocol and a Quant Studio 3 instrument (Applied Biosystems). PCR conditions, validation experiments, controls, and analysis are described in Supplementary materials.

### Northern analyses

Northern analyses were performed as previously described (45,46) employing the oligonucleotides described in Supplementary Table S3.

### Statistical analyses

Statistical analyses were performed using the GraphPad Prism 4 software. All data are expressed as mean ± SEM. Differences were analyzed by two-tailed unpaired *t* test (comparing only two experimental groups) or one-way ANOVA (when comparing more than two experimental groups) where *P* values of <0.05 (*) was considered significant. Each experiment was performed with at least biological triplicates.

## RESULTS

### Endogenously expressed Crm1-GFP forms nuclear export complexes with tRNA^Ile^_UAU_ *in vivo*

Previous cytological, genetic, and biochemical data supported the hypothesis that Crm1 functions in tRNA nuclear export (45,49). However, the data did not rule out the possibility that Crm1 functions indirectly in this process. Crm1 is a member of the Ran-dependent β-importin family; therefore, to function as a *bona fide* tRNA nuclear exporter, Crm1 must bind tRNA *in vivo* in a RanGTP-dependent process. To assess the ability of Crm1 to form such tRNA nuclear export complexes, we employed a modified version of a previously described *in vivo* co-immunoprecipitation (co-IP) assay (38,46). Our previous procedures assessed co-IP of tRNAs with nuclear exporters that were encoded by multi-copy plasmids. However, since over-production of Crm1 causes growth defects (Supplementary Figure S1), we redesigned the procedures to conduct the co-IP assay with endogenous levels of Crm1. The yeast cells utilized possess a gene replacement of *CRM1* with Crm1 tagged with GFP at the C-terminus (*CRM1-GFP*) which was obtained from the GFP-tagged yeast collection (50). To secure or prevent formation of exportin-tRNA complexes, the cells were transformed with plasmids encoding either galactose-regulated RanGTP (Gsp1-G21V)-locked or RanGDP (Gsp1-T24N)-locked mutant constructs, as previously described (38,46); transient galactose induction proceeded for 1 h. Hydrolysis of RanGTP to RanGDP leads to the dissociation of nuclear exportins from their bound cargos. Therefore, exportins remain associated with their cargo when hydrolysis of RanGTP to RanGDP is inhibited. In contrast, when Ran is predominantly in the GDP bound form, formation of exportin-cargo complexes is inhibited (51). Wild type (WT) yeast cells without tagged Crm1 but expressing the galactose inducible RanGTP-locked mutant served as a negative control.

**Figure 1.**
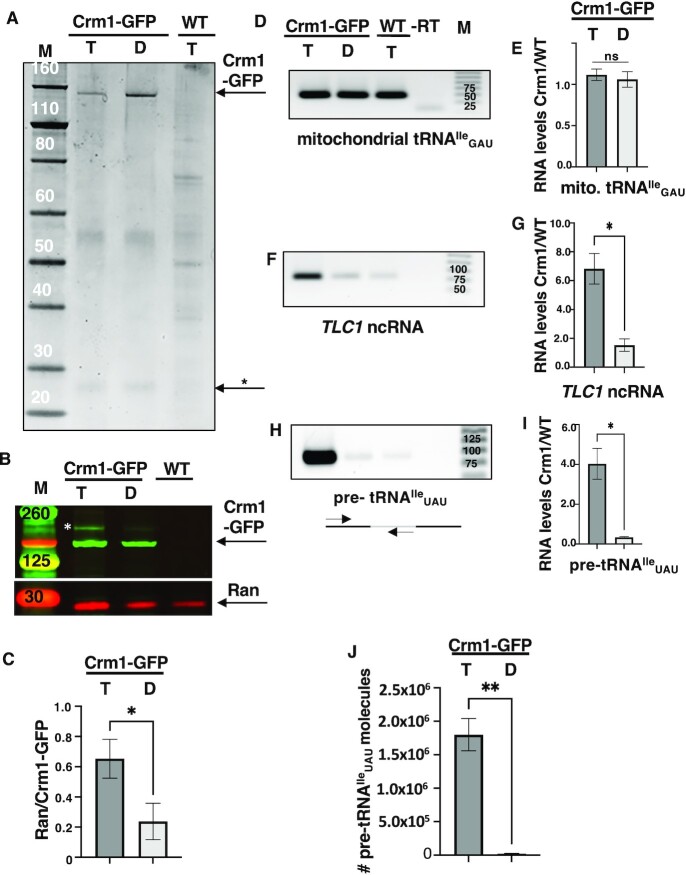
Endogenous levels of Crm1-GFP co-IP with intron-containing tRNA^Ile^_UAU_ *in vivo*. (**A**) Sypro Ruby staining of proteins enriched by co-immunoprecipitation employing anti-GFP. Lane 1: Yeast co-expressing Crm1-GFP and the RanGTP-locked mutant (Gsp1-G21V) ‘T’; lane 2: yeast cells co-expressing Crm1-GFP and RanGDP-locked mutant (Gsp1-T24N) ‘D’; lane 3: untagged wild type (WT) yeast cells expressing RanGTP-locked mutant. Putative Ran protein (*). Lane M: molecular size markers in kilodaltons. (**B**) Enriched levels of Crm1-GFP and Ran were detected by western blot analyses using anti-GFP or anti-Ran. Lanes 1, 2, 3 and M are as for A. Crm1-GFP protein indicated by the white asterisks likely resulted from incomplete uncrosslinking. *Note*: Panels A and B are separate gels. (**C**) Plot of the levels of Ran/Crm1-GFP in yeast cells expressing Crm1-GFP and either Ran-GTP locked (‘T’) or Ran-GDP-locked constructs (‘D’). (Panels D, F, H) RT-PCR analyses of RNAs co-purifying with Crm1-GFP. (**D**) mitochondrial tRNA^Ile^_GAU_, utilizing primers KC107 and KC108 for 40 cycles; (**F**) *TLC1* ncRNA using primers KCO57 and KCO58 for 30 cycles; and (**H**) intron-containing tRNA^Ile^_UAU_ using primers IVY3 and IVY1 for 27 cycles (Supplementary Table S2, Supplementary materials). Nomenclature is as for panel A. Lane -RT, no reverse transcriptase control. Lane M: DNA ladder in base-pairs. (Panels: **E**, **G**, **I**) Plots of the ratios of the signal of the amplified RNAs in Crm1 with Ran-GTP locked or with Ran-GDP-locked constructs/WT corresponding to panels D, F and H, respectively. All data are represented as mean ± SEM and were obtained for at least three biological replicates. *P < 0.05; ns = not statistically significant. (**J**) Level of intron-containing tRNA^Ile^_UAU_ co-IP with endogenous levels of Crm1-GFP transiently co-expressing RanGTP-locked ‘T’ or RanGDP-locked ‘D’ mutants, as determined by RT-qPCR. *Note*: RT-qPCR values do not represent absolute numbers of pre-tRNA molecules because tRNA modifications may partially block or slow the RT reactions. Experiments were conducted on three independently grown biological cultures. Data in (J) are represented as mean ± SEM. Statistical significance for intron-containing tRNA^Ile^_UAU_ pull-down results by RT-qPCR studies between the experimental groups was calculated using students unpaired *t*-tests; **P < 0.01.

Extracts from formaldehyde cross-linked cells expressing endogenous Crm1-GFP and co-expressing either of the mutant galactose-regulated Ran constructs were co-immunoprecipitated using anti-GFP-conjugated magnetic beads (38,46,52). The pull-down fractions were divided into two pools, one for protein and the other for RNA analyses. Reversal of the formaldehyde cross-links by heating the pools at 95°C for 30 min, followed by protein separation on polyacrylamide gels. Sypro-Ruby staining of the pull-down protein fractions revealed enrichment of a protein with the anticipated molecular weight of Crm1-GFP (Figure 1A). We verified the enriched protein to be Crm1-GFP by western analyses of immunoprecipitated proteins using anti-GFP (Figure 1B). As predicted, there was also co-enrichment of Ran as determined by employment of anti-Ran (Gsp1) (kind gift from Dr P. Belhumeur, Univ. Montreal). In contrast, somewhat lower levels of Ran co-purified with Crm1-GFP from cells containing Ran in the GDP-locked form or from WT cells expressing only the RanGTP-bound mutant construct (Figure 1B,C).

The RNA fractions were analyzed by RT-PCR (Figure 1D–I). As a negative control, we first assessed levels of nonspecific RNAs co-purifying with Crm1-GFP from each of the extracts. We reasoned that since Crm1 is a nuclear–cytoplasm shuttling protein not known to be associated with the mitochondrial matrix that tRNAs encoded by the mitochondrial genome would serve as negative controls for nonspecific co-purifying RNAs. To detect low levels of the mitochondrial RNAs, RT-PCR reactions were amplified for 40 cycles. We detected rather equivalent low levels of mitochondrial tRNA^Ile^_GAU_ in each of the pull-down fractions, *i.e*. from cells with Crm1-GFP and Ran in the GTP-locked form or cells with Ran in the GDP-locked form, and from WT cells with untagged Crm1 and the RanGTP-locked construct. The data document that each of the co-IP fractions contain little, but equivalent, levels of nonspecific RNAs (Figure 1D, E). For a positive control, we amplified *TLC1* ncRNA (30 cycles) in the pull-down fractions. Previous RNA immunoprecipitation studies documented physical interactions of Crm1 with *TLC1* ncRNA in yeast (53). We detected *TLC1* ncRNA in the pull-down fractions from Crm1-GFP cells containing the RanGTP-locked mutant, but not from cells containing the RanGDP-locked mutant, nor from WT cells expressing GTP-locked Ran (Figure 1F, G). The data document appropriate Crm1 exporter-RNA nuclear export complexes in our co-IP fractions.

To assess the relative amounts of intron-containing tRNA^Ile^_UAU_ forming nuclear export complexes with Crm1, we employed a reverse primer with sequence complementarity to the tRNA^Ile^_UAU_ intron and a forward primer binding to the 5′ exon (Figure 1H). RT-PCR amplification (27 cycles) detected unspliced tRNA^Ile^_UAU_ co-enriched in co-IP fractions from Crm1-GFP cells expressing GTP-locked Ran (Figure 1H, I). In contrast, RT-PCR analyses of co-IP of unspliced tRNA^Ile^_UAU_ from cells expressing Crm1-GFP and GDP-locked Ran or WT cells expressing only GTP-locked Ran, possessed exceedingly low levels of unspliced tRNA^Ile^_UAU_ (Figure 1H, I). These pull-down experiments were performed for three independent biological cultures with nearly identical results. In parallel, RT-qPCR analyses from the same RNA fractions were performed. The data (Supplementary Table S1; Figure 1J) confirm *in vivo* co-enrichment of intron-containing tRNA^Ile^_UAU_ with Crm1-GFP. In sum, these *in vivo* studies of endogenously expressed Crm1-GFP co-purifying with intron-containing tRNA^Ile^_UAU_ provide strong support for the hypothesis that Crm1 serves as a tRNA nuclear exporter.

### tRNA family preferences exhibited by Los1, Mex67, and Crm1 tRNA nuclear exporters

We reported that Los1 and Mex67 exhibit tRNA family preferences in the primary tRNA nuclear export step (46). Moreover, a recent biochemical assay demonstrated that Crm1 and Mex67, but not Los1 or Msn5, function in the re-export step for spliced tRNA^Phe^_GAA_ (49). Given that Crm1 is a *bona fide* tRNA nuclear exporter, the question arose whether it also may have preferences for particular tRNA families in primary tRNA nuclear export.

Previously, we employed northern analyses to assess the consequences of mutant Los1 and Mex67-Mtr2 proteins upon accumulation of various intron-containing pre-tRNA families. In those studies, accumulation of intron-containing pre-tRNAs served as a proxy for the efficiency of the primary tRNA nuclear export step. By those analyses we concluded that Los1 could export all 10 of the intron-containing pre-tRNAs to the cytoplasm; however, Los1 appeared to serve marginal roles in nuclear export of tRNA^Phe^_GAA_ and tRNA^Ser^_CGA_ (38,46). In contrast, Mex67-Mtr2 seemed to prefer tRNA^Ile^_UAU_, tRNA^Pro^_UGG_, tRNA^Trp^_CCA_ and tRNA^Tyr^_GUA_ as nuclear export cargo (46).

**Figure 2.**
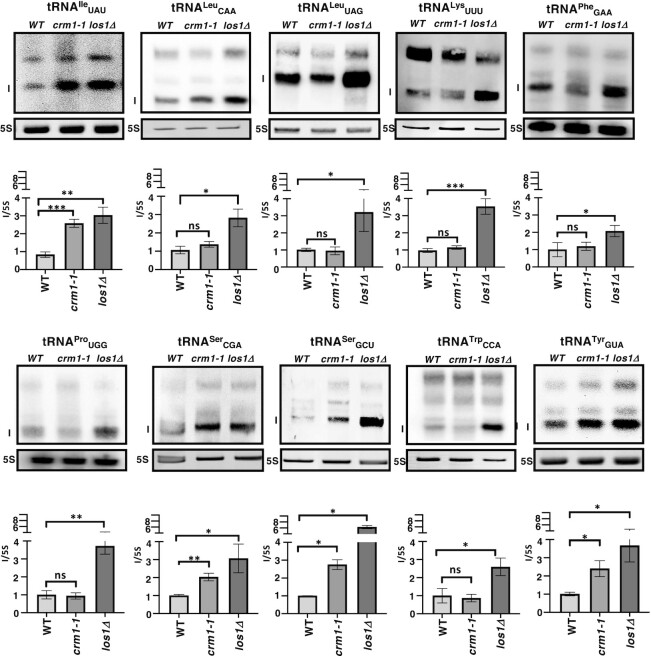
Family preference accumulation of end-processed, intron-containing pre-tRNAs from mutant *crm1-1* and *los1Δ* yeast cells. Small RNA isolated from log-phase wild-type (WT), *crm1-1* and *los1Δ y*east cells grown in YEPD rich media at 23°C and shifted to 37°C for 2 h were resolved on 10% polyacrylamide 7M urea gels and transferred to membranes. The tRNAs were detected using the tRNA specific probes, as indicated (probe sequences are provided in Supplementary Table S3). The ratios of the signal intensities of the 5′, 3′ end-processed intron-containing pre-tRNAs (I) were normalized to 5S rRNA levels (I/5S) in the same samples and then the relative level of I/5S in the mutants to WT was plotted with I/5S for WT set to 1. The RNAs migrating slower than the I band are pre-tRNAs prior to processing of 5′ and/or 3′ leader/trailer sequences. Note: For panel tRNA^Ser^_GCU,_ the lanes were spliced together due to the order on the gel being different from the other panels. All data are represented as mean ± SEM and were obtained for at least three biological replicates. *P < 0.05; **P < 0.01; ***P < 0.001; ns = not statistically significant.

To address possible Crm1 tRNA family cargo preferences, we conducted northern analyses similar to those conducted for Los1 and Mex67-Mtr2 (45,46). We compared levels of end-processed, intron-containing pre-tRNA for each of the 10 families of tRNAs encoded by intron-containing genes from *crm1-1* cells to levels of the same RNAs extracted from WT and *los1Δ* cells (Figure 2, RNA bands labeled I). RNAs were obtained from cell cultures grown in rich media and incubated for 2 h at 37°C (non-permissive temperature for *crm1-1*). The RNAs were resolved on 10% polyacrylamide 7M urea gels and transferred to membranes; intron-containing tRNAs were detected using the probes provided in Supplementary Table S3 and the levels of the RNAs labeled ‘I’ were normalized to 5S RNA levels in the same samples (Figure 2). Consistent with our previous report (45), we found that at the nonpermissive temperature *crm1-1* cells accumulate end-processed, intron-containing tRNA^Ile^_UAU_ and tRNA^Tyr^_GUA_. Interestingly, upon assessing the levels of all 10 end-processed, intron-containing tRNAs, we learned that pre-tRNA^Ser^_CGA_ and pre-tRNA^Ser^_GCU_ were also elevated in *crm1-1* cells compared to WT cells; however, the levels of the remaining 6 families of end-processed, intron-containing pre-tRNAs were not statistically elevated in *crm1-1* cells compared to WT cells. Thus, employing accumulation of end-processed, intron-containing tRNAs as a proxy for tRNA nuclear export, we conclude that Crm1 has preference to serve as a nuclear exporter for 4 of the 10 tRNA families encoded by intron-containing tRNAs. However, the conclusion that a tRNA is not a cargo if the mutant exporter does not demonstrate accumulation of end-processed, intron-containing tRNA must be tempered by the fact that in budding yeast there are at least three tRNA nuclear exporters and under the conditions employed (elevated temperature/rich media) particular tRNAs may be preferred and exported efficiently by the other exporters such that accumulation in the *crm1-1* mutant may not be detected.

To assess tRNA cargo preferences of the three tRNA nuclear exporters by an alternate and more direct means, we employed our co-IP procedures to compare the ability of the three exporters to assemble into tRNA nuclear export complexes with each of the 10 families of tRNAs encoded by intron-containing genes. Although by northern analyses Mex67-Mtr2 appeared to possess tRNA cargo preferences for tRNA^Ile^_UAU_, tRNA^Pro^_UGG_, tRNA^Tyr^_GUA_ and tRNA^Trp^_CCA_, over-expression of Mex67-Mtr2 substituted for Los1, leading to efficient export of all intron-containing tRNAs in *los1Δ* cells (46). Because over-expression apparently blurs tRNA substrate preferences of Mex67-Mtr2, we altered the strategies to conduct assembly of tRNA nuclear export complexes employing only endogenously expressed GFP-tagged nuclear exporters.

First, we conducted studies with yeast cells obtained from the yeast GFP-tagged gene replacement collection (50) to learn whether employment of endogenously expressed levels of Los1-GFP and Mex67-GFP, like Crm1-GFP, could detect tRNA nuclear export complexes. Since Los1 is a karyopherin, we also introduced plasmids that transiently co-expressed RanGTP-locked or GDP-locked mutants in the yeast strains. Western blot analyses of the co-IP extracts revealed enrichment of Los1-GFP and Mex67-GFP (Supplementary Figure S2A, lanes 4–6, red arrows). As expected, Ran co-purified with the Los1-GFP samples when Ran was locked in the GTP bound state, but there was sparce copurification of Ran with Los1-GFP in samples with Ran locked in GDP bound state or in the Mex67-GFP pull down fractions (Supplementary Figure S2A, compare lanes 5 and 6, yellow arrow). RT-PCR assays (27 cycles) documented co-enrichment of unspliced tRNA^Ile^_UAU_ from cells encoding endogenous levels of Mex67-GFP and Los1-GFP co-expressing RanGTP-locked mutant, but not Los1-GFP co-expressing RanGDP-locked mutant (Supplementary Figure S2B). The data document the efficacy of the pull-down assays for pre-tRNAs from cells with endogenously expressed Los1-GFP and Mex67-GFP nuclear exporters. Control experiments similar to those described above for Crm1 were also conducted to assure that the GFP-tagged exportins interacted appropriately with known RNA substrates. Thus, we documented that Mex67-GFP, but not Los1-GFP, co-IP with *PGK1* mRNA (30 cycles) (Supplementary Figure S2C). We also documented that each pull-down fraction contained very low but nearly equivalent amounts of nonspecific mitochondrial tRNA^Ile^_GAU_ (40 cycles) (Supplementary Figure S2D). The experiments were performed for three independent biological cultures and levels of unspliced tRNA^Ile^_UAU_ amplified by PCR were normalized to the background nonspecific mitochondrial tRNA^Ile^_GAU_ levels for each tRNA nuclear exporter; statistical significances between the experimental groups were calculated using students unpaired t-tests (Supplementary Figure S2E). The studies documented that our co-IP methodology can assess assembly of tRNA nuclear export complexes by all three endogenously expressed tRNA nuclear exporters, Los1, Mex67 and Crm1.

**Figure 3.**
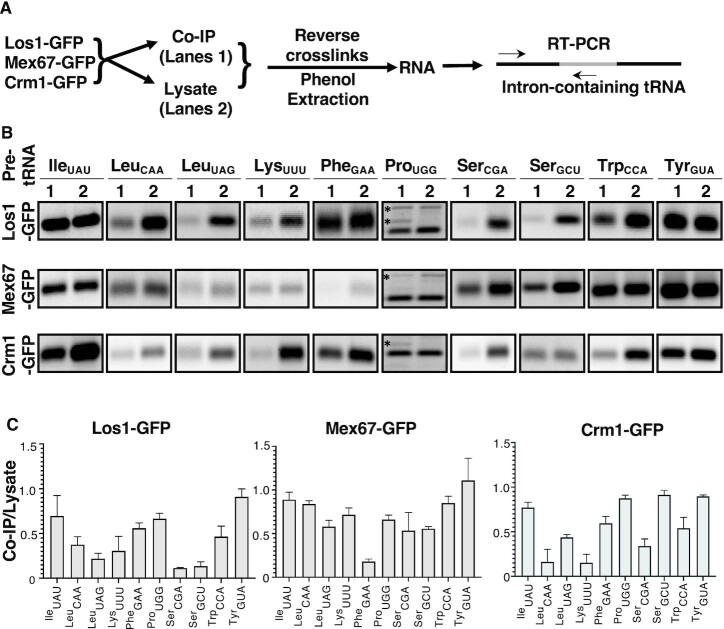
Los1, Mex67, and Crm1 exhibit tRNA family preferences for assembly into tRNA nuclear export complexes. (**A**) Strategy to assess tRNA family preference by tRNA exporters. Cells containing endogenously expressed Los1-GFP, Mex67-GFP or Crm1-GFP were cross-linked with formaldehyde and enriched via anti-GFP immunoprecipitation. RNA fractions obtained from co-IP fractions (lanes 1), or the lysates (lanes 2) were reverse cross-linked by heating and phenol extracted (Supplementary materials). RT-PCR assays were performed (27 cycles) with the RNA fractions employing primers indicated by arrows and detailed in Supplementary Table S2. Ten sets of primers were employed; for each set, the reverse primers were complementary to the particular tRNA intron sequences (gray box). PCR followed RT using forward primers corresponding to the 5′ exons (left black box) for each tRNA and the same reverse primer used in the RT reaction. (**B**) RT-PCR amplification of tRNAs in cell lysates or co-enriched with individual tRNA exporters. The specific pre-tRNAs are indicated above each panel. (**C**) Plots of the RT-PCR co-IP/lysate ratios. For each tRNA family, the RT- PCR levels of each intron-containing pre-tRNA family, co-enriched with the specified tRNA nuclear exporter was normalized to the RT-PCR levels of the same pre-tRNA in the lysate. The 10 intron-containing pre-tRNA families are indicated along the X-axis. Specific tRNA nuclear exporters are indicated above each bar graph. Note for the 10 tRNA^Pro^_UGG_ genes there are different intron sequences; the primer set utilized recognizes the intron for tP(UGG) located on chromosome XV. *, denotes unknown products which may be due to amplification of the other isoforms of pre-tRNA^Pro^_UGG_ introns. Plots of the RT-PCR co-IP/lysate are the ratios for the mean for three biological replicates ± SEM.

To assess the tRNA preferences of each of the endogenously expressed nuclear exporters, co-IP and lysate fractions obtained from each were analyzed by RT-PCR using primers corresponding to tRNA introns and 5′ exon sequences for each of the ten intron-containing tRNA family members (Figure 3A). The PCR-amplified intron-containing tRNAs that co-purified with the individual tRNA nuclear exporters were resolved on agarose gels and their levels were determined by measuring the band intensities (Figure 3B, lanes 1). Using the same primer sets, the tRNAs were also amplified by RT-PCR on the same amount of total RNA obtained from the cell lysates used for the pull-down assays (Figure 3B, lanes 2). The co-IP/Lysate RT-PCR ratios, representing the fraction of each intron-containing tRNA family member enriched by each of the three nuclear exporters compared to the total levels of the same tRNAs in the lysate were calculated and plotted to obtain a profile of the relative affinities of a particular tRNA nuclear exporter for each of the ten intron-containing tRNA families (Figure 3C).

The northern analyses were conducted with RNAs from yeast cultures grown at 23°C in rich media with glucose as the carbon source and shifted to 37°C for 2 h before harvesting. In contrast, the co-IP studies were conducted with RNAs from cells grown at 30°C in defined media with raffinose as the carbon source and shifted to galactose as the carbon source (to induce the Ran mutant constructs) 1 h before harvesting. Differences in environmental conditions could alter the levels of the exporters and/or their subcellular distributions (54) or the transcription/processing/location of particular tRNA families (55) and hence, affect the apparent cargo preferences of the tRNA exporters. Despite the caveats, the results of the northern versus co-IP methodologies possess similarities and the analyses revealed new insights. First, by both assays, certain intron-containing tRNA families such as pre-tRNA^Ile^_UAU_ and pre-tRNA^Tyr^_GUA_ are efficient cargos for all three tRNA nuclear exporters. Second, results from both assays suggest that the tRNA nuclear exporters exhibit tRNA family preferences that are distinct from one another and thus, the different tRNA nuclear exporters appear to cooperate with each other for the efficient nuclear export of particular intron-containing pre-tRNA families. Some of the same tRNA preferences were evidenced by both methodologies. For example, for Crm1, tRNA^Ile^_UAU_, tRNA^Ser^_GCU_ and tRNA^Tyr^_GUA_ are preferred cargoes by both assays and tRNA^Leu^_CAA_ and tRNA^Lys^_UUU_ are poor cargoes by both assays (compare Figures 2 and 3; Supplementary Table S4). In contrast, tRNA^Phe^_GAA_ and tRNA^Trp^_CAA_ are preferred Crm1 cargo only as assessed by the co-IP assay. Comparisons of tRNA^Pro^_UGG_ preferences are not relevant because the RT-PCR amplification assessed only 1 of the isodecoders whereas the northern analyses assessed all of the isodecoders. For Mex67-Mtr2, more RNA families are generally preferred as assessed by the co-IP assay (Figure 3) than by northern analysis (46). For Los1, tRNA^Phe^_GAA_ is a poor cargo by the northern analysis, but it is readily detected by the co-IP studies (compare Figures 2 and 3; also, (46); in contrast, by co-IP tRNA^Ser^_CGA_ and tRNA^Ser^_GCU_ are poor cargo for Los1 (compare Figures 2 and 3). Thus, even though the two methodologies—accumulation of end-processed, intron-containing tRNAs and co-IP of exporters with tRNAs—were conducted with cells grown in different types of media, different carbon sources, and incubated at different temperatures, there was concordance by both methodologies for several, but not all, of the tRNA family preferences. In sum, we suggest that the three yeast tRNA nuclear exporters possess tRNA family preferences that may vary under differing environmental conditions.

### Mex67-GFP, but not Los1-GFP nor Crm1-GFP, forms nuclear export complexes with 5′ leader-containing pre-tRNAs

Los1 preferentially binds to and exports appropriately structured tRNA with processed 5′ and 3′ termini to the cytoplasm (6,36,37,39). However, there is evidence that tRNAs can be exported to the cytoplasm prior to 5′ leader removal (34,35). Thus, we assessed the ability of the newly discovered tRNA nuclear exporters, Mex67 and Crm1, to form nuclear export complexes with 5′ leader-containing tRNAs.

**Figure 4.**
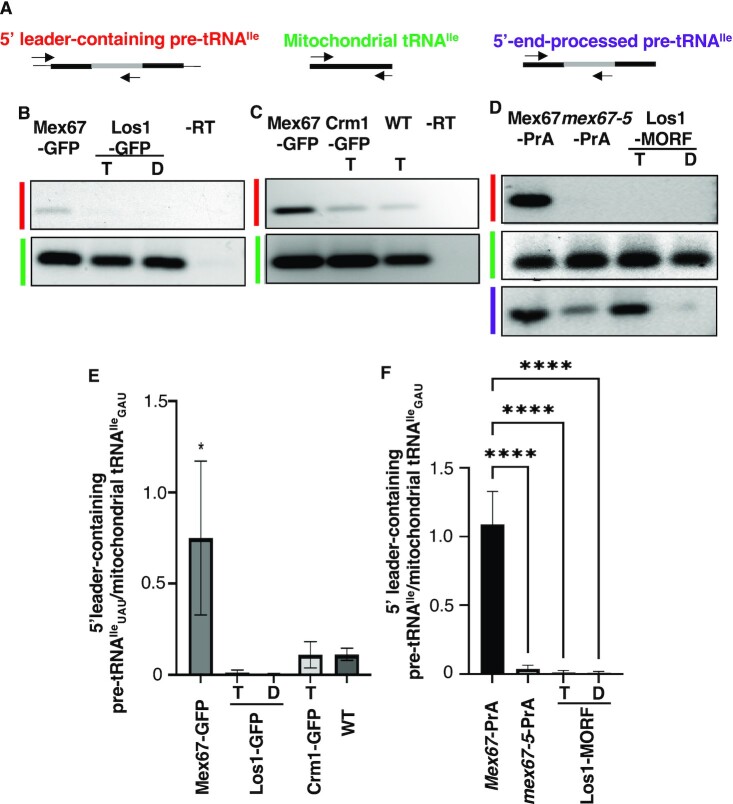
Endogenous and over-expressed Mex67-GFP co-IP with tRNA^Ile^_UAU_ containing the 5′ leader. (**A**) Illustration of the strategy to amplify RNAs via RT-PCR. Thin lines on either end of the drawing represent the 5′ leader and 3′ trailer sequences. Black boxes represent the exons, and the gray box represents the intron. Arrows depict forward and reverse primers. Colored text indicates the particular RNAs that were amplified; (**B**-**D**) RT-PCR: Red bars depict 5′ leader-containing unspliced tRNA^Ile^_UAU_, using a forward primer binding to 5′ leader sequence and first 3 bases of the 5′ exon of tRNA^Ile^_UAU_ (EK16) and reverse primer complementary to tRNA^Ile^_UAU_ intron (IVY3) (27 cycles). Green bars depict mitochondrial tRNA^Ile^_GAU_ (primers KC058 and KC107; 40 cycles). Purple bar indicates 5′ end-processed intron-containing tRNA^Ile^_UAU_ (27 cycles); -RT lane: No reverse transcriptase controls. (**B**) Co-IP with endogenous levels of Mex67-GFP or Los1-GFP co-expressing RanGTP-locked protein ‘T’ or RanGDP-locked ‘D’ mutant proteins; (**C**) Co-IP with endogenously expressed Mex67-GFP, Crm1-GFP co-expressing RanGTP-locked ‘T’, and wild-type cells co-expressing RanGTP-locked protein ‘T’; (**D**) Co-IP with over-expressed Protein A (PrA)-tagged Mex67, PrA-tagged mutant *mex67-5*, or over-expressed MORF-tagged Los1 transiently co-expressing RanGTP-locked or RanGDP-locked mutants; (E, F) Graphical representations of the RT-PCR experiments illustrating that only Mex67-GFP is able to bind pre-tRNA^Ile^_UAU_ containing 5′ leader sequences. (**E**) is a graph of data from endogenously expressed exporters [see panels (A) and (B)]; (**F**) is a graph of data from over-expressed exporters (see panel D). Y axis denotes the ratio of the band intensities of 5′ leader-containing pre-tRNA^Ile^_UAU_ to the band intensities of mitochondrial tRNA^Ile^_GAU_ obtained from RT-PCR reactions performed on pull-down fractions of specific exporters. tRNA nuclear exporters are indicated along the X-axis. Experiments were conducted on three independent biological cultures. All data are represented as mean ± SEM. Differences were analyzed by one-way ANOVA followed by post-hoc test; *P < 0.05; ****P < 0.0001.

Employing primer sets to amplify 5′ leader-containing unspliced tRNA^Ile^_UAU_ (27 PCR cycles) (Figure 4A; Supplementary Table S2) (35), we detected co-IP of tRNA^Ile^_UAU_ containing the 5′ leader sequence with Mex67 (Figure 4B,C, top panels). In contrast, there was very little co-enrichment of 5′ leader-containing tRNA^Ile^_UAU_ with Crm1-GFP or Los1-GFP (Figure 4B,C, top panels). Even cells with ∼5-fold over-expressed MORF-tagged Los1 (38,56) failed to bind pre-tRNA^Ile^_UAU_ with the 5′ leader sequence (Figure 4D, top panel). In contrast, Protein A-tagged Mex67, which expresses at 5-fold increased levels (46) robustly co-enriched with 5′ leader-containing pre-tRNA^Ile^_UAU_ (Figure 4D, top panel).

We performed additional controls to document the specificity of the co-IP assays. First, over-expressed Protein A-tagged *mex67-5* mutant protein, used as a negative control, had low co-IP of 5′ leader-containing pre-tRNA^Ile^_UAU_ (Figure 4D, top panel). Second, employing a forward primer for 5′ processed tRNA^Ile^_UAU_, we detected co-IP of both over-expressed Protein A-tagged Mex67 and MORF-tagged Los1 (Figure 4D, bottom panel), mimicking previous observations (46). Finally, as described above for endogenously expressed Crm1-GFP, we assessed mitochondrial-encoded tRNA^Ile^_GAU_ (40 PCR cycles) to monitor nonspecific co-purifying RNAs. Similar levels of background mitochondrial tRNA^Ile^_GAU_ were present in all samples (Figure 4B, C, bottom panel; 4D, middle panel).

The experiments for both the endogenously expressed (Figure 4E) and over-expressed (Figure 4F) exporters were performed in three independent biological replicates and the ratios of the band intensities for 5′ leader-containing pre-tRNA^Ile^_UAU_ versus mitochondrial tRNA^Ile^_GAU_ were plotted (Figure 4E, F). The data document that, among the three yeast tRNA nuclear exporters, only Mex67 forms levels of nuclear export complexes with 5′ leader-containing tRNA^Ile^_UAU_.

### 3′ to 5′ RNases turnover of aberrant 5′ leader-containing spliced tRNA^Ile^_UAU_

**Figure 5.**
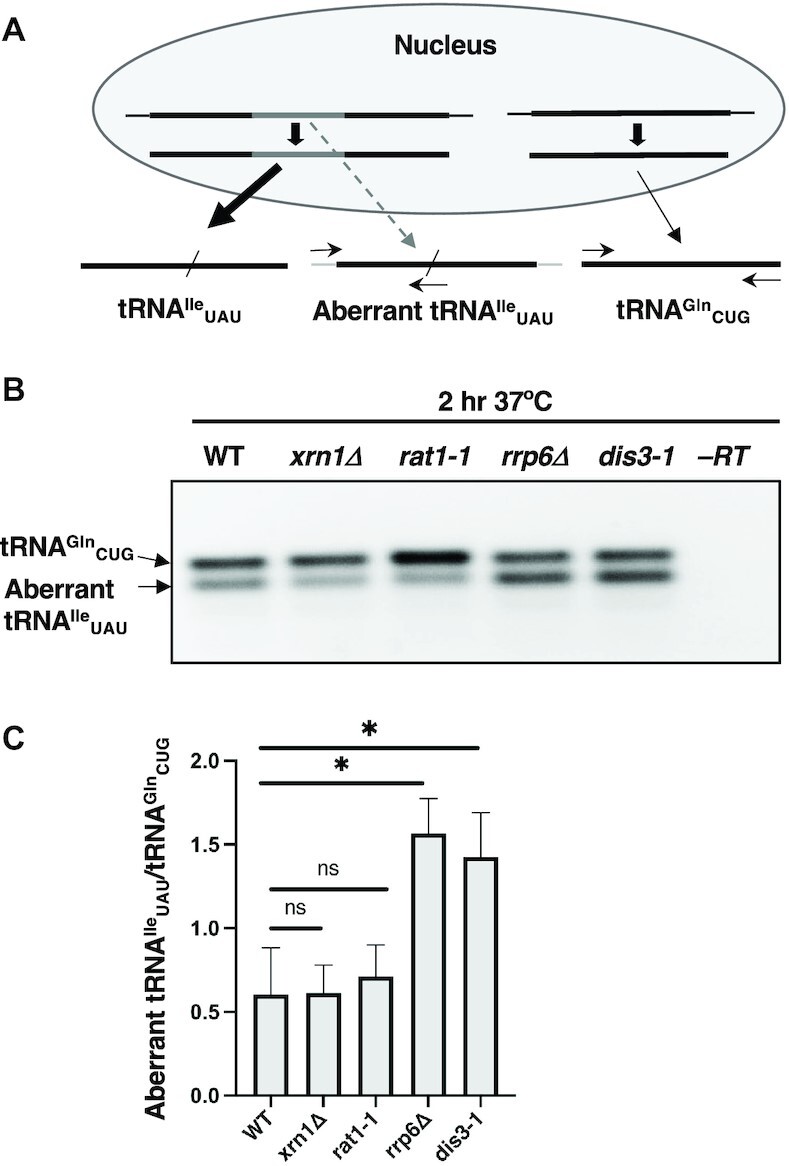
Spliced tRNA^Ile^_UAU_ containing the 5′-leader sequence accumulates in *rrp6*Δ and *dis3-1* mutant cells. (**A**) Strategy to detect 5′ leader-containing, spliced aberrant tRNA^Ile^_UAU_ by RT-PCR. Left: tRNA^Ile^_UAU._ Thin gray lines indicate the 5′ leader and 3′ trailer; thick black boxes indicate the exons; the thick gray box indicates the intron; the slash denotes the splice junction. Precocious nuclear export of the tRNA^Ile^_UAU_ primary tRNA transcript followed by splicing on yeast mitochondrial surface, indicated by the dotted down arrow, generates the aberrant spliced tRNA^Ile^_UAU_ containing the 5′ leader. Primers (EK16 and EK24) used in RT- PCR reactions to detect 5′ end-extended, spliced tRNA^Ile^_UAU_ are depicted. Right: RT-PCR for mature tRNA^Gln^_CUG_, employed as an internal control, is encoded by an intronless gene and was amplified (Primers EK100 and EK101) in the same reactions as tRNA^Ile^_UAU_. (**B**) RT-PCR analyses of RNA isolated from WT, *xrn1*Δ, *rat1-1*, *rrp6*Δ, or *dis3-1* yeast cells incubated at 37°C (the nonpermissive temperature for *rat1-1* and *dis3-1*) for 2 h. Lane -RT: No reverse transcriptase control. (**C**) Ratios of band intensities of 5′ leader-containing, spliced tRNA^Ile^_UAU_ to mature tRNA^Gln^_CUG_ for RNAs in the indicated yeast strains. All data are represented as mean ± SEM and were obtained for at least three biological replicates; *P < 0.05; ns = not statistically significant.

5′ Leader-containing spliced tRNA^Ile^_UAU_ (‘aberrant tRNA’) accumulates in cells in which retrograde tRNA transport is defective, presumably because tRNA retrograde import in WT cells functions in quality control for appropriate tRNA end-processing (35). However, it is unknown whether tRNAs with unprocessed 5′ termini that are imported into the nucleus are repaired and join the pool of functional tRNAs and/or if they are destroyed by nucleases. To study the possible turnover of these aberrant 5′ leader-containing spliced tRNAs, we determined the consequences of mutations of genes encoding RNA nucleases, including those involved in the rapid tRNA decay (RTD) pathway (57) and the RNA exosome (58). We employed RT-PCR using a forward primer complementary to the 5′ leader and the first three nucleotides of the 5′ exon and a reverse primer spanning the splice junction to amplify the aberrant tRNA, as previously described (35). In the same reaction, tRNA^Gln^_CUG_ was co-amplified as a loading control (35) (Figure 5A). For each mutant, the ratios of the intensity of the 5′ leader-containing, spliced tRNA^Ile^_UAU_ (Figure 5B, bottom bands) to the mature tRNA^Gln^_CUG_ (Figure 5B, top bands) were quantitated and compared to the ratio for WT cells (Figure 5C).

Four ribonucleases were investigated: two members of the 5′ to 3′ RTD RNases, nuclear Rat1 and cytoplasmic Xrn1, and two members of the RNA exosome: nuclear Rrp6 and nuclear and cytoplasmic Dis3. Upon isolation of RNAs from cells incubated for 2 h at 37°C (the nonpermissive temperature for *rat1-1* and *dis3-1* cells), neither *xrn1Δ* nor *rat1-1* mutants accumulated higher levels of aberrant tRNAs than that observed for wild-type cells (Figure 5B, C). In contrast, there was statistically significant accumulation of aberrant tRNA^Ile^_UAU_ from *rrp6Δ* and *dis3-1* mutant yeast cells (Figure 5B, C). The data indicate that aberrant tRNAs are turned over by 3′ to 5′ nucleases, but not by the 5′ to 3′ RTD nucleases. The data are especially interesting because although the exosome resides in both the nucleus and the cytoplasm, the unessential Rrp6 subunit is exclusively a component of the nuclear exosome (58). Since Rrp6 is a resident of the nucleus, turnover of the aberrant tRNAs must therefore occur after nuclear import of the spliced tRNA; however, the data do not eliminate the possibilities of turnover also occurring in the cytoplasm or repair in the nucleus. Thus, the tRNA retrograde pathway cooperates with the 3′ to 5′ quality control nucleases to eliminate aberrant spliced tRNAs that contain 5′ leader sequences that were generated by precocious tRNA nuclear export by Mex67-Mtr2 before 5′ leader removal.

## DISCUSSION

tRNA nuclear-cytoplasmic trafficking is a complex process comprised of three movements of tRNA molecules between the nucleus and the cytoplasm: primary nuclear export, retrograde nuclear import, and tRNA re-export. We previously demonstrated that the mRNA exporter, Mex67-Mtr2, co-functions with the canonical tRNA nuclear exporter, Los1, to export tRNAs to the cytoplasm (45,46). In this work, we document that the β-importin Crm1 expressed in endogenous levels also forms tRNA nuclear export complexes *in vivo* and it is thus a *bona fide* 3^rd^ tRNA nuclear exporter in budding yeast.

Export of tRNAs from the nucleus by Los1 is evolutionarily conserved. Following our report showing that Mex67-Mtr2 heterodimer also functions as a tRNA nuclear exporter in yeast (46), Hegedusova *et al.* reported that the Trypanosomatid Mex67 homologue, TbMex67, is involved in the nuclear export of a subset of tRNAs bearing the queuosine modification at position 34 whereas TbMtr2 exported all tested tRNAs (33) and Chen *et al.* documented that the Mex67 homologue, NXF1, functions in tRNA nuclear export in human cells (59). Therefore, it appears that tRNA nuclear export and substrate preferences by Mex67-Mtr2 may be evolutionarily conserved. In contrast, studies in *Xenopus* oocytes (60) and human cancer cells (59) found no role for the Crm1 homologue, Xpo-1, in tRNA nuclear export. Another study identified budding yeast Dbp5, a member of the DEAD box family of proteins, to function in tRNA nuclear export, most probably by collaboration with Mex67 and/or Crm1 (61). It will be interesting to determine the roles for Mex67-Mtr2 and Crm1 homologues in tRNA nuclear export in various other eukaryotic organisms.

It is unclear whether the identification of Crm1 as the 3^rd^ tRNA nuclear exporter completes the picture of tRNA nuclear export in budding yeast. Despite the abundance of information acquired by our genome-wide screen identifying the gene products involved in the tRNA nuclear export process, the northern hybridization screen assessed only a single tRNA, tRNA^Ile^_UAU_ (45). It is fortuitous that tRNA^Ile^_UAU_ was utilized as tRNA^Ile^_UAU_ is a cargo preferred by all three tRNA exporters by both the northern and the co-IP assays. However, since yeast tRNA nuclear exporters possess tRNA family preferences, it is possible that there are yet unidentified tRNA nuclear exporter(s) which do not export tRNA^Ile^_UAU_ efficiently and hence would not have been uncovered in the genome-wide screen.

The canonical tRNA nuclear exporter Los1 binds to tRNA structural features that are in common for all tRNAs (36,37,39). Thus, it was predicted that Los1 exports all tRNA substrates with equal efficiency from the nucleus. Also, over-expression of the Mex67–Mtr2 heterodimer resulted in suppression of nuclear accumulation of all 10 intron-containing pre-tRNA families in *los1Δ* cells (46), leading to the prediction that all 10 intron-containing tRNAs also would be efficiently exported by Mex67–Mtr2. However, surprisingly, our data support the hypothesis that the three tRNA nuclear exporters possess tRNA family preferences. The implications for each of the exporters having tRNA family preferences could be far-reaching as translation of the proteome could be affected by alteration of the cellular balance/activities of individual tRNA exporters. Thus, it will be interesting to determine whether the tRNA nuclear exporters or the pre-tRNAs are differently expressed or differently located in cells under various environmental conditions.

The question arises: what features render one intron-containing pre-tRNA family a preferred substrate for a given tRNA nuclear exporter over the others? For Mex67-Mtr2 and Crm1-mediated tRNA nuclear export, tRNA preferences are likely due to protein adaptors. Crm1 interacts with proteins containing the Leu-rich motifs (62) and does not possess a RNA binding domain. So, Crm1 must employ an RNA binding adapter protein to bind tRNA cargo. Although the metazoan Mex67-Mtr2 heterodimer homologue, NXF1-NXT1, can directly bind particular RNAs (63–65), it generally interacts with mRNAs via protein adaptors (62,66). Thus, features recognized for binding pre-tRNA cargoes may be due to adapter proteins that facilitate binding to tRNA cargoes. Since the availability/regulation of the putative adapters could regulate the roles of Crm1 and/or Mex67-Mtr2 in tRNA nuclear export, it is important that they are identified and characterized to gain a more complete understanding of tRNA export. The possibility also remains that nucleoside modifications catalyzed in the nucleus on particular intron-containing tRNA families influence their direct or adapter-mediated binding with tRNA exporters.

As tRNAs function iteratively in translation, there needs to be quality control of the delivery of appropriately processed and structured tRNAs to the cytoplasm. Biochemical and structural studies have shown that vertebrate and *S. pombe* Los1 homologues participate in tRNA quality control by binding to tRNAs that are end-processed and appropriately structured (36,37,39). Here, we report that budding yeast Los1 also does not bind tRNA^Ile^_UAU_ containing 5′ leaders to detectable levels even when over-expressed. Despite such strict quality control by Los1, tRNAs with 5′ leaders have been detected in yeast cells (34,35). The co-IP data reported here document that Mex67, but not Crm1, binds 5′ leader-containing tRNA^Ile^_UAU_, exporting pre-tRNA^Ile^_UAU_ to the cytoplasm before appropriate leader/trailer maturation. The finding that Crm1 does not form nuclear export complexes with leader-containing tRNAs was somewhat unexpected since the Crm1 homologue, Xpo-1, can export microRNAs that contain 5′ extensions (67). The lack of an obvious yeast homolog of PHAX, the adapter protein associated with Xpo-1 that binds 5′ end-extended U snRNAs (68), may provide an explanation for this difference. It also underscores how adapter proteins may dictate substrate preference among nuclear exporters. Also, as addressed above, it will also be important to learn how modifications of the intron-containing tRNAs influence their *in vivo* interactions with each of the three tRNA nuclear exporters and which of the three nuclear exporters/adaptors inappropriately deliver the levels of hypomodified tRNAs to the cytoplasm that we previously detected (35).

Cells possess mechanisms to prevent cytoplasmic accumulation of improper tRNAs. The tRNA retrograde pathway that removes aberrant tRNAs from the cytoplasm and delivers them to the nucleus provides one of these mechanisms (35). Once the 5′ leader-containing spliced aberrant tRNAs return to the nucleus, nuclear exosome components, such as Rrp6 and Dis3, can eradicate these aberrant tRNAs. It is equally possible that, after returning to the nucleus, the aberrant tRNAs are repaired and given a second chance to become functional tRNAs because the enzymes necessary to process 5′ and 3′ ends are localized in the nucleus. These turnover and repair processes may not be mutually exclusive. Thus, components of the tRNA retrograde pathway and the nuclear exosome provide layers of tRNA quality control in yeast.

Ohira and Suzuki (34) reported that 5–20% of individual pre-tRNA families are capped; however, they were unable to measure the level of capped endogenous tRNA^Ile^_UAU_. Since Mex67 is primarily a nuclear exporter for mRNA, it is possible that a pool of the pre-tRNAs pulled down by Mex67 is capped. We have not tested this possibility because the pulled-down total RNA fraction would not differentiate whether the cap belongs to mRNA or tRNA co-immunoprecipitating with Mex67.

Interestingly, nuclear and cytoplasmic exonucleases of the RTD pathway, Rat1 and Xrn1, which degrade RNAs in the 5′ to 3′ direction appear not to be involved in the quality control of 5′ leader-containing tRNA. It is possible that these leader-containing tRNAs are capped or that they bear 5′ triphosphates, so that Xrn1 would not be able to degrade them. In fact, LC/MS analyses demonstrated the absence of 5′ monophosphate on 5′ leader-containing pre-tRNA^Ile^_UAU_ (34), a prerequisite for Rat1/Xrn1 activities. Capped tRNAs with 5′ extensions have been detected for tRNAs other than tRNA^Ile^_UAU_ (34). It would be interesting to learn whether like for tRNA nuclear export, there is a tRNA family-specific mode for tRNA turnover mechanisms as well.

In sum, we report that there are at least three exporters that co-function in tRNA nuclear primary nuclear export: Los1, Mex67-Mtr2, and Crm1. Surprisingly, each of the tRNA nuclear exporters has preferences for particular tRNA cargoes, possibly linking tRNA nuclear export to environmental conditions and to the regulation of protein synthesis. This work also demonstrates the interplay between tRNA nuclear trafficking and the tRNA quality control pathways to ensure functional cytoplasmic pools of tRNAs.

## DATA AVAILABILITY

All data to support the conclusions in this study have been made available either within the paper or in the supplemental data file.

## Supplementary Material

gkac754_Supplemental_File

## Notes

Present address: Kunal Chatterjee, Department of Biology, Wittenberg University of Springfield, OH 45504, USA.
