# Supplementary material for: Three tRNA nuclear exporters in S. cerevisiae: parallel pathways, preferences, and precision
Source: Nucleic Acids Res. 2022 Sep 13;50(17):10140–52. doi: 10.1093/nar/gkac754 (PMC9508810; doi:10.1093/nar/gkac754)
Supplement: gkac754_Supplemental_File [file gkac754_supplemental_file.pdf]

Chatterjee *et al*. Supplementary Materials
Contains:

- **Supplementary Methods**
- **Tables S1-S4**
- **Supplementary Figures and legends.**

SUPPLEMENTARY METHODS

In vivo co-immunoprecipitation assay:

Yeast cells co-expressing GFP-tagged Crm1 from its endogenous promoter and galactose inducible Ran-locked mutants were grown in SC-LEU media and incubated at 30°C to OD_{600} of 0.3-0.4. Additon of galactose (2% final concentration) iwas carried out for 1 hr prior to harvesting. In parallel, BY4741 cells expressing RanGTP-locked mutant were grown under the same conditions. Chemical cross-linking of RNA-protein complexes was carried out by formaldehyde addition to the cultures [final concentration 0.3%]. After 30', the cross-linking was quenched by glycine addition solution [final concentration of 66 mM for 10 min}. Harvested cells were rapidly frozen in liquid nitrogen and cryolyzed, using a planetary ball mill (Alber et al. 2007). 0.5 mg of frozen, ground cells were suspended in 5 ml of RNP-compatible extraction buffer (20 mM HEPES, pH 7.4, 110 mM KOAc, 40μM $MgCl_2$,100 mM NaCl, 0.5% Triton, 0.1% Tween-20, 0.05 mM DTT, 0.24 μg/μl Heparin Sodium,1:1000 dilution of protease inhibitor cocktail set IV (EMD Millipore), 1:1000 dilution of Solution P, 1:1000 dilution of Antifoam A (Sigma) and 1:5000 dilution of RNase inhibitor (RNaseOUT, Invitrogen); 10 μM of GTP were added to the extracts of Crm1-GFP and BY4741 cells that contained RanGTP-locked constructs. After centrifugation at 3000xg at 4°C for 10 min, the soluble extract was clarified by passing through a 1.6 μ GD/X glass microfiber syringe filter (25 mm) (Whatman). The lysate was then incubated with GFP-conjugated magnetic beads at 4°C for 30 min. The beads were then collected with a magnet, washed six times with 1 ml of ice-cold Wash buffer A (20 mM HEPES, pH 7.4, 110 mM KOAc, 40 μM $MgCl_2$, 0.5% Triton, 0.1% Tween-20, 0.05 mM DTT, 0.24 μg/μl of Heparin Sodium,1:1000 dilution of protease inhibitor cocktail set IV (EMD Millipore), 1: 1000 dilution of Solution P, 1: 1000 dilution of Antifoam A and 1:5000 dilution of RNase inhibitor (RNaseOUT)) and once with ice-cold Wash Buffer B (0.1 M NH_4OAc, 0.1 mM $MgCl_2$, 0.02% Tween-20). The beads, suspended in wash buffer B, were divided into two equal fractions.

One fraction was used for protein extraction, by incubating the beads with freshly prepared protein elution buffer (0.5 M NH_4OH, 0.5 mM EDTA) for 20 min at room temperature. The eluates were lyophilized overnight using a Speed Vac. The lyophilized pellets were suspended in LDS protein-loading buffer [Sample Buffer (4X)] containing lithium dodecyl sulfate (LDS) at pH 8.5 with SERVA Blue G250 and phenol red) with freshly added 50 mM DTT and heated at 95°C for 30 min to disassemble the putative complexes cross-linked by formaldehyde. After centrifugation at 2000 rpm for 90 sec, the protein suspensions were separated on a 4-12% NuPAGE Novex Bis-Tris precast gel (Life technologies) according to the manufacturer's specifications. A fraction of the proteins was stained with Sypro Ruby (Molecular Probes) according to the manufacturer's specifications. The remaining proteins were used for Western blot analyses.

To obtain enriched RNAs, the other half of the magnetic beads were suspended in wash buffer B and then were collected using a magnet and re-suspended in RNA elution buffer (50 mM Tris-HCl, pH 7.4, 10mM EDTA, 1% SDS, 10 mM DTT), followed by 70°C incubation for 45 min. The samples were then incubated with Proteinase K (20 mg/ml) (NEB) for 30' at 30°C. Enriched RNAs were extracted using an equal volume of acid phenol pH 4.4, then precipitated with 3 volumes of 100% EtOH and 0.5 μg of Glycogen co-precipitant (Ambion) and stored overnight at -80°C. RNA pellets obtained by centrifugation were re-suspended in nuclease-free water.

RT-PCR:

For RT-PCR analyses in Figs. 1 and 4, enriched RNAs from co-immunoprecipitation were treated with Turbo DNase (Ambion) following manufacturer's protocol. First strand cDNA was synthesized using SuperScript III reverse transcriptase (Invitrogen) following the manufacturer's protocol. PCR reactions were carried out using 1 μl of cDNA with GoTaq Flexi PCR system (Promega). The PCR conditions were as follows: For unspliced pre-$tRNA^{Ile}_{UAU}$: 2 min at 95°C, 27 cycles of 30 sec at 95°C, 20 sec at 52°C, and 20 sec at 72°C; 30 sec at 72°C; 2 picomoles of primers (IVY1 and IVY3 pre-$tRNA^{Ile}_{UAU}$). For mitochondria encoded $tRNA^{Ile}_{GUA}$, 2 min at 95°C, 35 cycles of 30 sec at 95°C, 20

sec at 43°C, and 20 sec at 72°C; 30 sec at 72°C;10 picomoles of primers KC107 and KC108. For TLC1 ncRNA**,** 2 min at 95°C; 30 cycles of 30 sec at 95°C, 20 sec at 57°C, and 20 sec at 72°C; 2 picomoles of primers KC057 and KC058. 15 µl of PCR reactions were resolved by electrophoresis on 2% agarose gels.

For experiments in Fig. 3, 10 ng of immunoprecipitated total RNA fraction were used for RT-PCR assays. In parallel, 10 ng of total RNA extracted from the lysates used in pull-down assays were also used to ascertain endogenous tRNA level in each strain. 2 picomole of reverse primer complimentary to intron of individual tRNAs were used for reverse transcriptase reactions with Superscript III enzyme, following manufacturer's protocols. 1 ml of cDNA was subsequently used for PCR reactions using a forward primer specific for 5' exon and the same reverse primer used in RT-reaction for individual tRNAs. Primer sequences are detailed in Table S2. PCR reactions were: 2 min at 95°C; 27 cycles of 30 sec at 95°C, 20 sec at 55°C, and 20 sec at 72°C; 30 sec at 72°C.

For experiments in Fig. 5, in which $tRNA^{Ile}_{UAU}$ and $tRNA^{Gln}$ levels were simultaneously assessed, the concentrations of each of the primer sets were previously optimized (Kramer and Hopper 2013), to allow for visualization of DNA resulting from amplifications of high- and low-abundance tRNAs on a single gel. 2 picomoles each of primers EK24 (complementary to splice junction of $tRNA^{Ile}_{UAU}$) and EK101 (complementary to 3' end of $tRNA^{Gln}_{CUG}$) were used in RT reactions. First strand cDNAs were synthesized using SuperScript III reverse transcriptase (Invitrogen) following manufacturer's protocol, with a starting amount of 100 ng of DNase treated RNA from each strain. PCR reactions were carried out using 1 µl of cDNA with GoTaq Flexi PCR system (Promega). The PCR reactions included 10 picomoles of forward (EK16) and reverse primers (EK24) specific to $tRNA^{Ile}_{UAU}$ and 2 picomoles of forward (EK100) and reverse primers (EK101) specific to $tRNA^{Gln}_{CUG}$. Primer sequences are detailed in Table S2. The PCR conditions are: 2 min at 95°C; 27 cycles of 30 sec at 95°C, 20 sec at 49°C, and 20 sec at 72°C; 30 sec at 72°C. 15 µl of the PCR products were resolved on a 3% Agarose gels at 100 V for 4 hr.

RT-qPCR:

The PCR conditions were as follows: a) Hold Stage: 2 min and 30 sec at 50°C, 20 sec at 95°C; b) PCR Stage: 40 cycles of 1 sec at 95°C, 20 sec at 60°C; c) Melt curve stage: 1 sec at 95°C, 20 sec at 60°C, 1 sec at 95 °C. No template controls were analyzed for primer sets and no RT controls were analyzed for each sample. Melt curve analysis verified specific amplification of the target. To prepare standard curves for unspliced $tRNA^{Ile}_{UAU,}$ tRNA species were reverse transcribed from RNA extracted from lysates used in the pull-down, followed by PCR using the primer sets specific for 5' exon and the intron of $tRNA^{Ile}_{UAU}$. PCR products were separated on gel and extracted using QIAquick Gel Extraction Kit (Qiagen). Gel extracted PCR products were then measured using Nanodrop Microvolume UV-Vis Spectrophotomer and standard curves were prepared with 10-fold serial dilutions of gel-extracted, RT-PCR products as templates to determine the concentrations of co-immunoprecipitated RNAs. PCR reactions using a forward primer specific for 5' exon and the same reverse primer used in RT-reaction for individual tRNAs. The primer pairs used in the RT-qPCR reactions was IVY1 and IVY3. Primer sequences are detailed in Table S2.

Supplementary References:

Alber F, Dokudovskaya S, Veenhoff LM, Zhang W, Kipper J, Devos D, Suprapto A, Karni-Schmidt O, Williams R, Chait BT et al. 2007. The molecular architecture of the nuclear pore complex. *Nature* **450**: 695-701.

Kramer EB, Hopper AK. 2013. Retrograde transfer RNA nuclear import provides a new level of tRNA quality control in Saccharomyces cerevisiae. *Proc Natl Acad Sci U S A* **110**: 21042-21047.

Table S1: RT-qPCR analyses of unspliced pre-tRNA$^{Ile}_{UAU}$ co-purifying with Crm1-GFP

	Crm1-GFP + RanGTP-locked (molecules)	Crm1-GFP + RanGDP-locked (molecules)
Unspliced tRNA$^{Ile}_{UAU}$	$1.8 \times 10^6 \pm 2.4 \times 10^5$	$1.9 \times 10^4 \pm 1.4 \times 10^4$

Number of pre-tRNA$^{Ile}_{UAU,}$ molecules co-immunoprecipitated with endogenous levels of Crm1-GFP transiently co-expressing RanGTP-locked (T) or RanGDP-locked (D) mutants, as determined by RT-qPCR. The numbers denote the absolute number of pre-tRNA$^{Ile}_{UAU}$ molecules pulled down by Crm1-GFP in the presence of Ran mutants normalized to mitochondrial tRNA$^{Ile}_{GAU}$ levels present in the same lysates. Experiments were conducted on three independent grown biological cultures. All data are represented as mean ± SEM.

Table S2: List of primers for PCR and RT-qPCR

RNA species detected	Primer pairs	Figure
Unspliced tRNA$^{Ile}_{UAU}$	IVY1 GCTCGTGTAGCTCAGTGGTTAG	Fig. 1, Fig. 3. Fig. 4, Fig. S2
	IVY3 cttttaaaggcctgtttgaaag	
TLC1 ncRNA	KC057 AAGCCTACCATCACCACACC	Fig. 1
	KC058 AAACAGCGAACTCGTGCAAA	
Mitochondrial tRNA$^{Ile}_{GAU}$	KC107 GAAACTATAATTCAATTGGTT	Fig. 1, Fig. 4, Fig. S2
	KC108 TGGTGAAACTAACAGG	
PGK1 mRNA	KC038 ATGTCTTTATCTTCAAAGTTGTCTGTCCAAG	Fig. S2
	KC039 GATCTTCTTACCGTCCAATGGGACG	
Unspliced tRNA$^{Leu}_{CAA}$	JW044 tattcccacagttaactgcggtcaagatattt	Fig. 3
	KC104 GGTTGTTTGGCCGAGCGGT	
Unspliced tRNA$^{Leu}_{UAG}$	KC101 GAGattttagaggttaaatccaCCT	Fig. 3
	KC102 GGGAGTTTGGCCGAGTGGT	
Unspliced tRNA$^{Lys}_{UUU}$	KC105 GTatccttgcttaagcaaatgcgctTA	Fig. 3
	KC106 TCCTTGTTAGCTCAGTTGG	
Unspliced tRNA$^{Phe}_{GAA}$	KC093 GATaacttgaccgaagtttttCT	Fig. 3

	GN08<br>CGGACTTAGCTCAGTTGG	
Unspliced $tRNA^{Pro}_{UGG}$	KC098<br>GGGCGTGTGGTCTAGTGGTA	Fig. 3
	KC113<br>tgctttgtcttcctgtttaatcaggaagtcg	
Unspliced $tRNA^{Ser}_{CGA}$	KC094<br>ATagccgaactttttattccaTTC.	Fig. 3
	KC095<br>GGCACTATGGCCGAGTGGT	
Unspliced $tRNA^{Ser}_{GCU}$	KC096<br>CCTaattgcttttctgaggaaaTA	Fig. 3
	KC097<br>GTCCCAGTGGCCGAGTGGT	
Unspliced $tRNA^{Trp}_{CCA}$	KC100<br>GAAGCGGTGGCTCAAT	Fig. 3
	KC099<br>ATtgcaatcttattccgtggaatttccaagatttaaTT	
Unspliced $tRNA^{Tyr}_{GUA}$	GN54<br>ttcgtagtgataaa	Fig. 3
	Ivy117<br>CTCTCGGTAGCCAAG	
Unspliced $tRNA^{Ile}_{UAU}$ with 5' leader	EK16<br>TCTTTCGAAAATGCT	Fig. 4, Fig. S2
	IVY03<br>cttttaaaggcctgtttgaaag	
Spliced $tRNA^{Ile}_{UAU}$ with 5' leader	EK16<br>TCTTTCGAAAATGCT	Fig. 5
	EK24<br>GTCGCGTTATAAGCA	
$tRNA^{Gln}_{CUG}$	EK100<br>GGTCCTATAGTGTAG	Fig. 5
	EK101<br>GGTCCCACCCGGATTC	

Sequences corresponding to introns are designated in lower case; sequences corresponding to exons are provided in upper case.

Table S3 Oligonucleotide probes employed for northern analyses

Name	Function	Sequence
JW48	$tRNA^{Ile}_{UAU}$	GGCACAGAAACTTCGGAAACCGAATGTTGCTATAAGCACGAA GCTCTAACCACTGAGCTACACGAGC
SM12	$tRNA^{Leu}_{CAA}$	GTTAACTGCGGTCAAGATATTTCTTGAATCAGGCGCCTTAGA CCG
SM13	$tRNA^{Leu}_{UAG}$	ATTTTAGAGGTTAAATCCACCTAAATCTGACGCCTTAAACC
SM15	$tRNA^{Lys}_{UUU}$	CCTTGCTTAAGCAAATGCGCTTAAAAGCCGAACGCTCTACC
SM10	$tRNA^{Phe}_{GAA}$	TAACTTGACCGAAGTATTTCTTCAGTCTGGCGCTCTCC
SM18	$tRNA^{Pro}_{UGG}$	CCCAAAGCGAGAATCATACCACTAGACCACACGCCC
SM20	$tRNA^{Ser}_{CGA}$	AGCCGAACTTTTTATTCCATTCGAGTCTCTCGCCTTAACCACT CGGCCATAGTGCC
SM21	$tRNA^{Ser}_{GCU}$	AATTGCTTTTCTGAGGAAATAGCAGGGCATCGCCTTAACCAC TCGGCCACTGGGAC
SM11	$tRNA^{Trp}_{CCA}$	CGTGGAATTTCCAAGATTTAATTGGAGTCGAAAGCTCTACC
KC031	$tRNA^{Tyr}_{GAU}$	CCCGATCTCAAGATTTCGTAGTGATAAATTACAGTCTTGCGC CTTAAACC

Red font corresponds to intron sequences; black font corresponds to 5' exon sequences; blue font corresponds to 3' exon sequences. There are alternative introns for $tRNA^{Pro}_{UGG}$ and the 5' exon probe hybridizes to all the intron-containing isodecoder pre-tRNAs.

Table S4 Comparison of data derived from northern and IP analysis for Los1 and Crm1

tRNA	Los1		Crm1	
	Northern I/5S *los1Δ* /I/5S WT Glucose 37°C 2 hr.	Co-IP Los1-GFP C0-IP/Lysate Raffinose + gal 1hr	Northern I/5S *crm1-1*/I/5S WT Glucose 37°C 2 hr.	Co-IP Crm1-GFP Co-IP/Lysate Raffinose + gal 1hr
$tRNA^{Ile}_{UAU}$	3.0	0.70	2.5	0.75
$tRNA^{Leu}_{CAA}$	2.8	0.35	ns	0.12
$tRNA^{Leu}_{UAG}$	3.2	0.20	ns	0.40
$tRNA^{Lys}_{UUU}$	3.5	0.30	ns	0.12
$tRNA^{Phe}_{GAA}$	2.0	0.55	ns	0.60
$tRNA^{Ser}_{CGA}$	3.0	0.10	2.0	0.30
$tRNA^{Ser}_{GCU}$	6.0	0.15	2.8	0.80
$tRNA^{Trp}_{CCA}$	2.5	0.50	ns	0.55
$tRNA^{Tyr}_{GUA}$	3.7	1.00	2.4	0.75

Blue highlighted: favored cargoes for Crm1 and Los1 by both northern and co-IP methodologies. Gray highlighted: unfavored cargoes for Crm1 by both northern and co-IP methodologies. Yellow highlighted: discordance between cargo preferences between the northern and co-IP methodologies for both Crm1 and Los1.

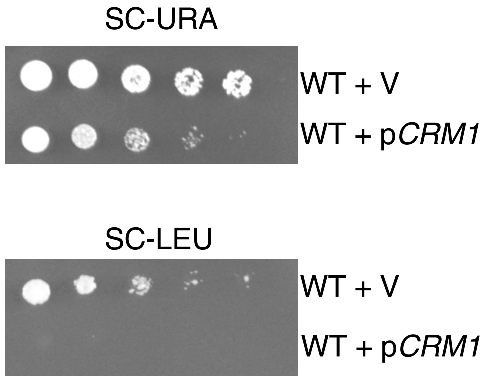

Fig. S1. Over-expression of Crm1 is detrimental to yeast cell growth. WT yeast cells transformed either with pYEX4T-1 plasmid alone (WT + V) or pYEX4T-1 containing *CRM1* with its endogenous promoter and terminator (WT + p*CRM1*) were grown to log phase and serially diluted. The cells were spotted on either SC-URA media or SC-LEU media and grown at 23°C for 3 days.

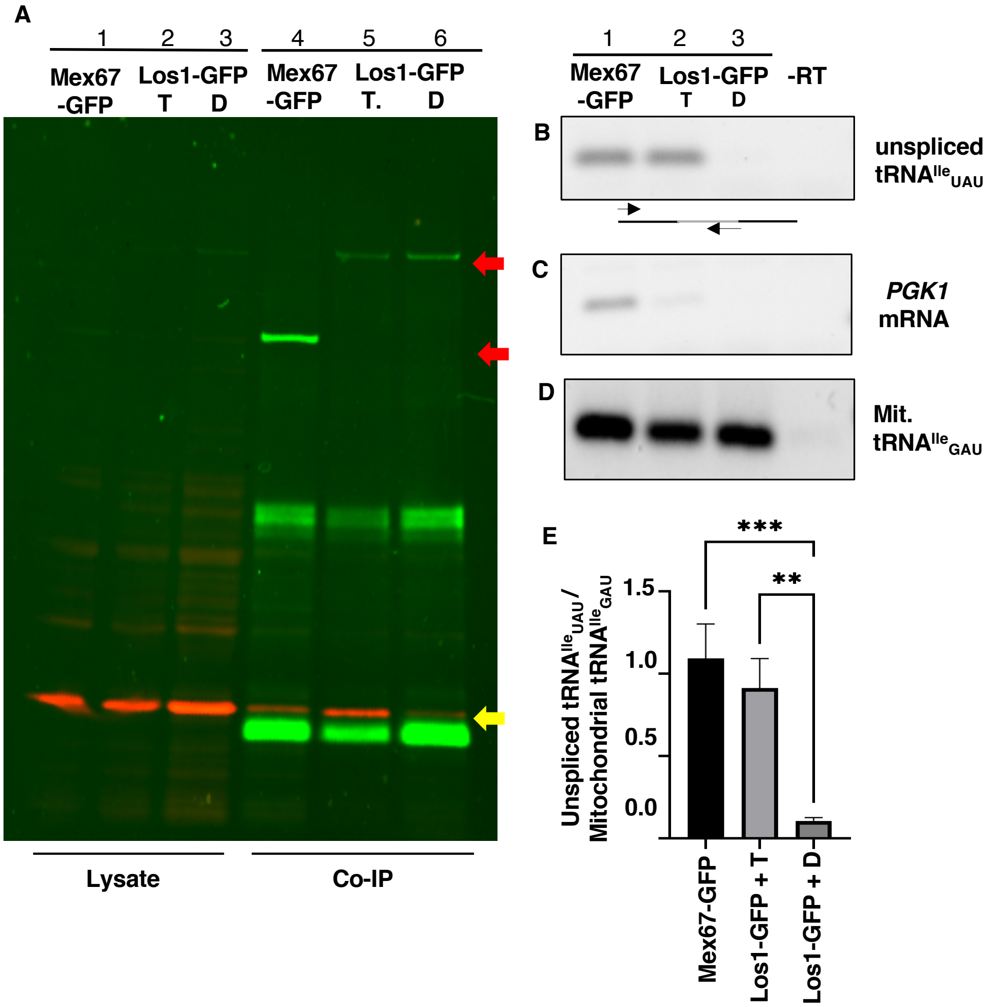

A
1 2 3 4 5 6
Mex67 -GFP
Los1-GFP T D
Mex67 -GFP
Los1-GFP T. D
Lysate
Co-IP
1 2 3
Mex67 -GFP
Los1-GFP T D
-RT
B
unspliced tRNAIle UAU
C
PGK1 mRNA
D
Mit. tRNAIle GAU
E
Unspliced tRNAIle UAU / Mitochondrial tRNAIle GAU
1.5
1.0
0.5
0.0
***
**
Mex67-GFP
Los1-GFP + T
Los1-GFP + D

Fig. S2. tRNAs co-purify with endogenous levels of GFP-tagged Los1 and Mex67. (A). Mex67-GFP and Crm1-GFP detected by Western Blotting. Lane 1: Un-crosslinked lysates of cells expressing endogenous levels of GFP-tagged Mex67. Lane 2: Un-crosslinked lysate of cells co-expressing endogenous levels of GFP-tagged Los1 and plasmid-encoded galactose-inducible RanGTP-locked mutant (T). Lane 3: Uncrosslinked lysate of cells co-expressing GFP-tagged Los1 from its endogenous locus and plasmid-encoded RanGDP-locked mutant (D). Lane 4: GFP-tagged Mex67 obtained by co-immunoprecipitation using anti-GFP. Lanes 5 and 6: Enriched levels of GFP-tagged Los1 co-expressing RanGTP or RanGDP-locked mutants, respectively. Enriched GFP-tagged tRNA nuclear exporter proteins and Ran are indicated by red and yellow arrows, respectively. (B-F). RT-PCR analyses of RNAs co-purifying with Mex67-GFP and Los1-GFP: (B) intron-containing $tRNA^{Ile}_{UAU}$; (C) *PGK1* mRNA; (D) mitochondrial $tRNA^{Ile}_{GAU}$. The strains corresponding to each lane are indicated on the top of panel (B) using the same nomenclature as for panel A. Lane -RT: Control reaction lacking reverse transcriptase. Schematic representations of oligonucleotides (arrows) to assess unspliced $tRNA^{Ile}_{UAU}$ are indicated below panel (B). Exons (Black lines); Intron (gray line). (E) Plot of the relative levels of pre-$tRNA^{Ile}_{UAU,}$ co-immunoprecipitated with endogenous levels of Mex67-GFP and Los1-GFP, transiently co-expressing RanGTP-locked (T) or RanGDP-locked (D) mutants, as determined by RT-PCR. Y-axis denotes the ratio of band intensities representing pre-$tRNA^{Ile}_{UAU}$ levels pulled down by Crm1-GFP in the presence of Ran mutants normalized to the band intensities representing pulled-down mitochondrial $tRNA^{Ile}_{GAU}$ levels. Experiments were conducted on three independent grown biological cultures. All data are represented as mean ± SEM. Differences were analyzed by two-tailed unpaired *t* test where *P* value of <0.05 was considered significant.
